# TLR3 activation by *Clonorchis sinensis* infection alleviates the fluke-induced liver fibrosis

**DOI:** 10.1371/journal.pntd.0011325

**Published:** 2023-05-11

**Authors:** Yuru Wang, Pengtao Gong, Xuancheng Zhang, Xiaocen Wang, Xu Zhang, Nan Zhang, Yanhui Yu, Yeting Ma, Haoyang Zhang, Xichen Zhang, Xin Li, Jianhua Li

**Affiliations:** State Key Laboratory for Zoonotic Diseases, Key Laboratory for Zoonosis Research of the Ministry of Education, Institute of Zoonosis, and College of Veterinary Medicine, Jilin University, Changchun, China; University of Liverpool, UNITED KINGDOM

## Abstract

*Clonorchis sinensis* is a zoonotic parasite associated with liver fibrosis and cholangiocarcinoma development. The role of toll-like receptors (TLRs) in *C*. *sinensis* infection has not yet been fully elucidated. Here, the TLR3 signaling pathway, cytokine expression and liver fibrosis were examined in *C*. *sinensis-*infected wildtype (WT) and *TLR3*^*-/-*^ mice. Polyinosinic-polycytidylic acid (Poly (I:C)) was used to treat *C*. *sinensis* infections. The results showed that TLR3 deficiency caused severe clonorchiasis with increased parasite burden, exacerbated proinflammatory cytokine expression and liver lesions, promoted the TGF-β1/Smad2/3 pathway and myofibroblast activation, exacerbated liver fibrosis (compared to WT mice). Poly (I:C) intervention increased the body weight, decreased mouse mortality and parasite burden, reduced liver inflammation, and alleviated *C*. *sinensis-*induced liver fibrosis. Furthermore, *C*. *sinensis* extracellular vesicles (CsEVs) promote the production of IL-6, TNF in WT biliary epithelial cells (BECs) via p38/ERK pathway, compared with control group, while TLR3 deletion induced much higher levels of IL-6 and TNF in *TLR3*^-/-^ BECs than that in WT BECs. Taken together, TLR3 inhibit IL-6 and TNF production via p38/ERK signaling pathway, a phenomenon that resulted in the alleviation of *C*. *sinensis*-induced liver fibrosis. Poly (I:C) is a potential treatment for clonorchiasis.

**Summary**

*Clonorchis sinensis* is a zoonotic parasite associated with liver fibrosis and cholangiocarcinoma development. Further understanding of the pathogenesis of *C*. *sinensis* especially liver fibrosis would help us develop a novel strategy for controlling clonorchiasis. Toll-like receptors (TLRs) serve as the host’s first line of defense against pathogens, however the role of TLRs in *C*. *sinensis* infection has not yet been fully elucidated. To elucidate the potential mechanism of TLR3 in *C*. *sinensis*-induced liver fibrosis, liver fibrosis modeling assays and cellular level assays *in vivo* and *in vitro* were performed using TLR3-deficient or normal C57/BL6 mice in our research. A new role for TLR3 in controlling *C*. *sinensis*-induced liver fibrosis was identified. TLR3 deficiency resulted in severe clonorchiasis increased parasite burden, exacerbated proinflammatory cytokine expression and liver lesions, promoted the TGF-β1/Smad2/3 pathway and myofibroblast activation, aggravated liver fibrosis. Poly (I:C) intervention alleviated liver inflammation and liver fibrosis in mice infected with *C*. *sinensis* and is a promising drug for clonorchiasis treatment. Our results will help in understanding the molecular mechanisms governing the host’s immune responses to *C*. *sinensis* infections and provide new information about clonorchiasis treatment.

## Introduction

*Clonorchis sinensis* is a zoonotic parasitic that is also known as the Chinese liver fluke. It inhabits the biliary system of humans and other piscivorous animals and can cause clonorchiasis [1]. Definitive hosts are infected by consuming under cooked or raw freshwater fish that contain metacercariae. Approximately 15 million people are infected by this liver flukes in East Asia, including over 13 million in China [2]. Infection is usually asymptomatic, although it may cause bile duct inflammation, hyperplasia, and fibrosis in chronically infected [1]. As it can cause cholangiocarcinoma (CCA) in chronic patients, *C*. *sinensis* has been classified as a class I carcinogen [3,4]. The recommended treatment for clonorchiasis is praziquantel; however, side effects such as mild or transient headaches, dizziness, nausea, abdominal discomfort, and drug resistance are unavoidable [1]. An effective vaccine for *C*. *sinensis* is currently unavailable [1,5]. Further understanding of the pathogenesis of *C*. *sinensis* especially liver fibrosis and CCA would help us develop a novel strategy for controlling clonorchiasis.

Toll-like receptors (TLRs) are the most potent initiators of the inflammatory response and serve as the host’s first line of defense against pathogens [6]. The TLR4-TGF-β/Smad signaling pathway regulates myofibroblast activation induced by *C*. *sinensis* [7]. The TLR2-regulated MAPK pathway and the release of reactive oxygen species regulate the *C*. *sinensis-*induced inflammatory response [8]. TLR9 recognizes CsEVs, promotes IL-6 and TNF release, and regulates inflammatory responses in clonorchiosis [9]. Evidence suggests that TLR3 is involved in the progression of liver fibrosis. Studies have confirmed that TLR3 is an important target protein that regulates Hepatic stellate cells (HSCs) activation and promotes liver regeneration. Polyinosinic-polycytidylic acid (Poly (I:C) (an agonist of TLR3) can significantly inhibit the activation of HSCs and block liver regeneration [10]. Moreover, TLR3 activation promotes the secretion of IL-10 and PGE2 by bone marrow mesenchymal stem cells (BMMSCs) to improve the therapeutic function of BMMSCs in alcoholic liver disease [11]. However, whether *C*. *sinensis* can activate the TLR3 pathway and the role of TLR3 in liver fibrosis caused by *C*. *sinensis* has not been extensively explored.

To investigate the role of TLR3 in *C*. *sinensis*-induced liver fibrosis, we established a mouse model of *C*. *sinensis*-induced liver fibrosis using wildtype (WT) and *TLR3*^*-/-*^ mice and examined various parameters associated with clonorchiosis, including weight, mortality, parasite burden, liver inflammation and biliary injury, cytokine expression, myofibroblast activation, TGF-β1 expression, phosphorylation level of Smad2/3, and intrahepatic collagen deposition. We also intervened *C*. *sinensis-*infected mice with poly (I:C) and evaluated the above indicators to understand the therapeutic effects of the TLR3 agonist on clonorchiasis caused by *C*. *sinensis*. To determine whether TLR3 and its downstream pathways regulate proinflammatory cytokine expression, mouse biliary epithelial cell (BECs) were stimulated with *C*. *sinensis* extracellular vesicles (CsEVs), and phosphorylation of p38, ERK, and p65, as well as cytokine expression, were examined. In addition, the mouse model of CsEVs injection was established to investigate the roles of CsEVs in regulating the activation of TLR3 and the contribution to biliary injuries.

## Materials and methods

### Ethics statement

All experimental procedures involving animals were conducted in compliance with Chinese legal standards, and the experiments were approved by the Animal Welfare and Research Ethics Committee of Jilin University. (IACUC permit number: 20160612).

### Animals

*TLR3*^*-/-*^ and WT female C57BL/6 mice (aged 6–8 weeks) were housed under constant temperature and pathogen-free animal conditions for 12 h on a dark/light cycle with sterile water and normal mouse chow.

### Collection of *C*. *sinensis* and CsEVs

The *C*. *sinensis* excretory/secretory proteins (CsESPs) were immediately used to isolate CsEVs [9]. The *C*. *sinensis* adults were isolated from bile duct of metacercariae infected WT C57BL/6 mice: The mice were euthanized after 35 days post infection (dpi) and disinfected with 75% alcohol for 15 min. Then, the mice were transferred to a sterile platform, and aseptic procedures were performed throughout the trial. Carefully isolate the liver, soak it in sterile PBS, and open the bile duct. Gently pressing the liver using ophthalmic forceps and picking out the adults.

The isolate adults were incubated at density of five parasites per mL of serum-free RPMI-1640 at 37°C for 6 h. The incubated supernatant was collected and centrifuged at 280×g and 2 000×g, continuously. Then the supernatant was filtered through a syringe filter (0.22-μm, Millipore, Massachusetts, USA). Then a slightly modified differential centrifugation method was used to collect CsEVs from the CsESPs [12]. Briefly, CsESPs were centrifuged at 10 000 ×g for 1 h, the supernatant was collected then passed through a filter (0.22 μm). The filtered liquid was centrifuged at 100 000 ×g for 1 h, and the sediment was collected. The precipitate was dissolved in sterile phosphate-buffered saline (PBS) and the CsEVs concentration was determined using BCA. Then, CsEVs on copper grids were negatively stained using carbon and 3% phosphotungsticacid and observed under a transmission electron microscope (TEM) (HITACHI, Tokyo, Japan). And a fraction of CsEVs were treated with RNase A (Solarbio, Beijing, China) to remove dsRNAs in CsEVs-free ESPs (rCsEVs).

### Isolation of mouse BECs

The biliary tree was obtained by removing liver membranes and hepatocytes through perfusion using HEPS solution (Biosharp, Anhui, China) (with 0.05mg/mL collagenase IV(Sigma-Aldrich, Missouri, US)) as conventional methods [13,14]. Then, the isolated biliary tree was divided into small pieces and further digested in 0.05mg/mL collagenase IV solution for 30 min under shaking conditions, and were centrifuged at 800 ×g for 10 min. The precipitate was retained, digested by 0.25% trypsin-digested (VivaCell, Shanghai, China) at 37° C for 10min. After centrifugation (800 ×g for 10 min), trypsin was discarded and RPMI-1640 medium with 5% serum was added to stop digestion. After repeated washing and centrifugation, the BECs were resuspended using RPMI-1640 medium (with 5% serum) and filtered through a 75μm aperture metal sieve. Cell number and viability were assessed using a 0.4% trypan blue solution (Procell, Wuhan, China) and BECs with 3 ×10^5^ were plated incubated at 37°C with 5% CO_2_ in 6 well tissue culture plates. To investigate CsEVs functions, The BECs were plated and treated with the CsEVs (50 μg/mL), rCsEVs (50 μg/mL) or PBS cultures for 2 h or 18 h at 37°C with 5% CO_2_, respectively.

### *C*. *sinensis*-caused liver fibrosis mouse model

*TLR3*^*-/-*^ and WT mice were inoculated with 200 *C*. *sinensis* metacercariae by gavage in 200 μL of PBS (pH 7.4), with mice administered 200 μL of PBS as the control. Poly (I:C)-intervened WT mice were injected intraperitoneally twice with poly (I:C) (10 mg/kg) (InvivoGen, California, US) at 0, 10, and 20 dpi. Weight and mortality rates were monitored daily until sacrifice. Mice were euthanized at 7, 15, and 35 dpi to examine the liver lesions and intrahepatic parasite burdens. Mouse faeces were collected daily after infection. Rice grain sized faeces were placed on a slide, added 50 μL of saline then covered with a coverslip to examine the eggs under the microscope. The liver tissues were isolated and used for cytokine detection, quantitative real-time PCR (RT-qPCR), western blotting, histological observation, Masson staining, and immunohistochemical staining.

### CsEVs injection

The mice of CsEVs group were intravenously injected with CsEVs (50 μg/mice, 50 μL) or PBS (50 μL) at the indicated time (1d, 3d,5d,7d) [15]. At the 8d, the mice were euthanized, and the livers were collected to detect TLR3 mRNA expression, cytokine secretion, liver lesions and collagen deposition.

### RT-qPCR analysis

Total RNAs from WT mouse livers and BECs was extracted using the TRIzol reagent and the cDNA was synthesized from the total RNA using reverse transcriptase prior to the PCR step. Then the mRNA levels were determined by RT-qPCR followed the conditions and procedures of Green qPCR Mix (Monad, Suzhou, China). The primer sequences used were TLR3 F: 5-AAGACAGAGACTGGGTCTGGG-3, R:5- AAGGACGCCTGCTTCAAAGT-3; and GAPDH F: 5-CCATGTTTGTGATGGGTGTG-3, R: 5-CCTTCTTG ATGTCATCATAC-3 [16]. The data were normalized to GAPDH and the mRNA level of all experimental groups was presented in terms of numerical vales calculated using (delta)(delta)C/t equation.

### Cytokine detection

Liver tissues of *TLR3*^*-/-*^ and WT mice were lysed into cell suspensions, and the supernatant was stored for cytokine detection. WT and *TLR3*^*-/-*^ mouse BECs were incubated with CsEVs (50 μg/mL) for 18 h, and poly (I:C) (30 μg/mL) was used as the positive control [17]. To investigate the role of p38 and ERK signaling pathways in regulating cytokine production, the WT BECs were pretreated with p38 or ERK inhibitors (Sigma-Aldrich, Missouri, US) for 60 min at 37°C, with untreated cells as the control. Then the cells were co-stimulated with CsEVs for 18 h. The secretion levels of cytokines in the liver tissues and BECs supernatant were detected using ELISA kits (IL-4/IL-6/IFN-γ/TGF-β1/TNF, Thermo Scientific, Massachusetts, US) [17].

### Histology observation

Liver tissues from the same positions were removed and embedded in paraffin. The tissue sections were sliced to a thickness of 3 μm, deparaffinized with xylene, and stained with hematoxylin and eosin. Hepatic injury and inflammation were thoroughly documented under a microscope and evaluated using the hepatic histological activity index (HAI) [18,19].

### Masson staining

The paraffin-embedded liver tissues were subjected to the same procedure until routine deparaffinization, and the tissue sections were stained with Masson’s Trichrome Stain Kit (Solarbio, Beijing, China). The positive area of collagen fibers was scanned and quantified using the Image-Pro Plus software (Media Cybernetics, Massachusetts, USA).

### Immunohistochemistry

Liver tissue was sliced into 5 μm sections and analyzed using routine immunohistochemistry. After peroxidase removal and antigen repair, the liver tissue sections were treated with FBS at room temperature for 30 min and then incubated overnight with CK-19 and α-SMA antibodies at 4°C, followed by incubation with HRP-conjugated antibodies. After counterstaining with hematoxylin, the tissue sections were mounted with neutral gum. The positive area of immunohistochemistry was digitized and analyzed using the Image-Pro Plus software. Antibody information is presented in S1 Table.

### Immunofluorescence

Adult *C*. *sinensis* was isolated, transferred to a cell culture dish, and incubated in 1640 medium for 12 h. After washing with sterile PBS, the worms were fixed and permeabilized, and dsRNAs was labeled with J2 antibody and FITC-labeled fluorescent secondary antibody. The nuclei were stained with Hoechst (Sigma-Aldrich, Missouri, US), and the subcellular localization of dsRNA was observed using the Live Cell Imaging System (Olympus, Tokyo, Japan). Antibody information is presented in S1 Table.

### Western blot

Liver tissues and BECs were collected and resuspended in RIPA lysis buffer containing PMSF (1:100) (Boster Bio, California, USA). The SDS-PAGE and membrane transfer tests were executed as previously reported [17]. The membranes were incubated overnight at 4°C with primary antibodies (Phosphorylated protein p65, ERK, Smad2/3, p38 and Total protein p65, ERK, Smad2/3, p38). Membranes were incubated with secondary antibody for 1 h at room temperature after three washes with PBST. An ECL-Chemiluminescence meter was used to visualize the protein (Clinx Science Instruments Co., Ltd., Shanghai, China). The protein expression level was quantified using ImageJ (National Institutes of Health, Bethesda, Maryland, USA) and the relative gray values of phospho-p65/GAPDH, phospho-Smad2/3/GAPDH, phospho-ERK/GAPDH and phospho-p38/GAPDH were calculated using Excel (Microsoft Corp., Redmond, WA, USA) software, respectively [9]. Antibody information is presented in S1 Table.

### Dot Immunobinding Assay (DIBA)

The dot immunoassay was performed according to the previous protocol [20]. Briefly, the sheared nitrocellulose membranes (NCM) were placed into reaction wells and 50 μL of CsEVs (1 mg or 0.1mg/mL), Poly (1:C) (30 μg/mL), and PBS were dropped onto NCM, and acted at room temperature for 30 min, and the remaining liquid was discarded. Then NCM was sealed with 5% milk, and then incubated overnight with J2 antibody at 4°C. The membrane was incubated at room temperature with HRP-link antibodies for 30 min after three washes with PBS, and the results were viewed using an imaging system. Antibody information is presented in S1 Table.

### Statistical analysis

GraphPad Prism Software (version 6.01) was used to conduct Tukey tests (T-tests) and two-way ANOVA on the data set and generated pictures (GraphPad Software Inc, California, US). The experiment data was obtained from three independent experiments, with the results expressed as the mean ± SEM. Significance was set at *p < 0.05, **p < 0.01, and ***p < 0.001.

## Results

### 1. TLR3 deficiency caused more severe clonorchiasis in *C*. *sinensis*-infected mice

**Fig 1:**
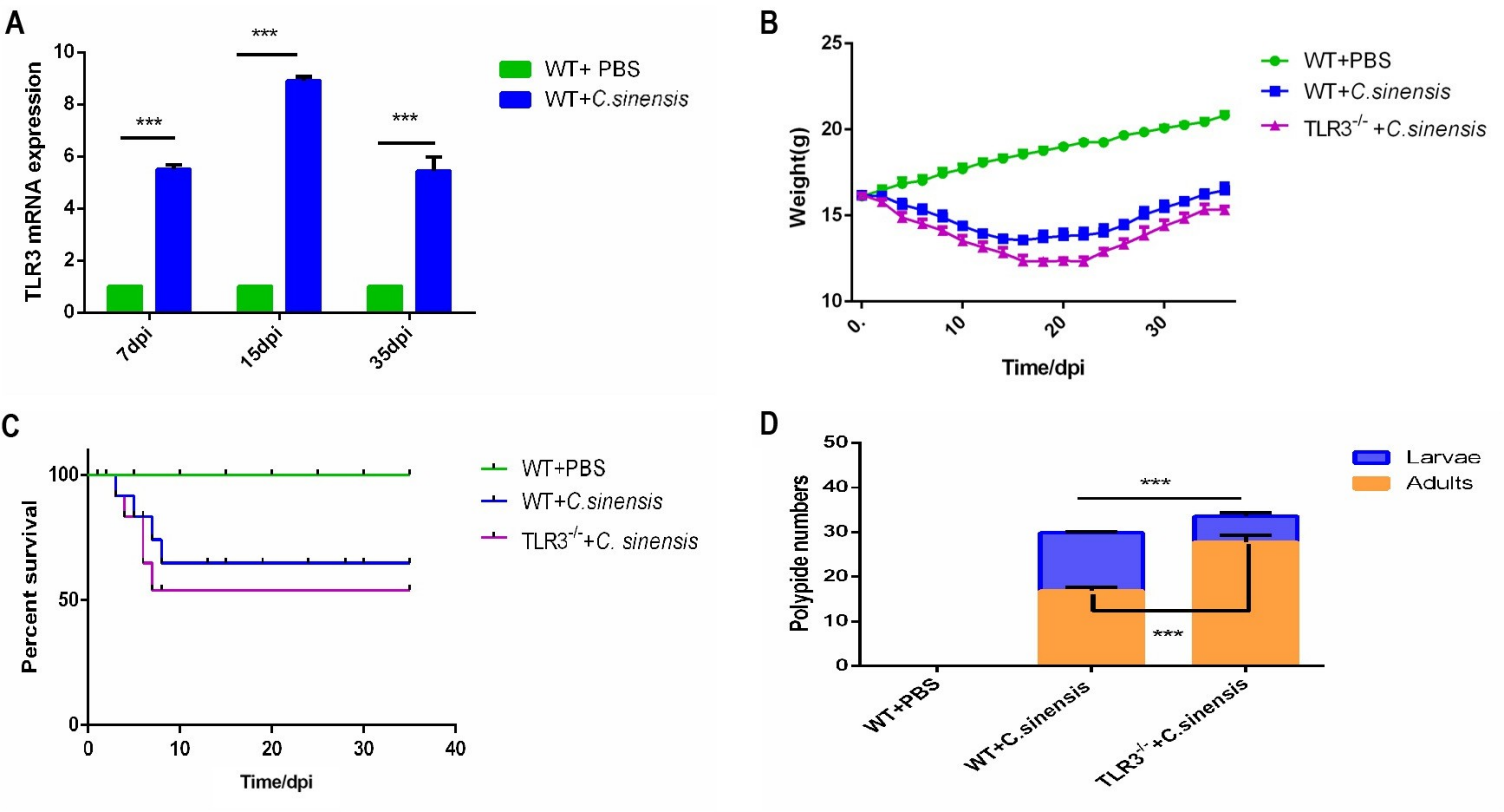
TLR3 deficiency caused severe clonorchiasis in *C*. ***sinensis*-infected mouse.** (A) TLR3 expression in *C*. *sinensis*-infected mouse liver. The transcription levels of TLR3 in liver of WT and *TLR3*^*-/-*^ mice were detected by RT-qPCR and normalized to GAPDH. (B) The weight changes of *C*. *sinensis*-infected WT and *TLR3*^*-/-*^ mice. The weight of *C*. *sinensis*-infected WT and *TLR3*^*-/-*^ mice were monitored daily and statistically plotted. PBS-treated WT mice served as negative control. (C) The percent survival of *C*. *sinensis*-infected WT and *TLR3*^*-/-*^ mice. The mortality of WT and *TLR3*^*-/-*^ mice were recorded daily and plotted after *C*. *sinensis* infection. (D) The numbers of intrahepatic parasites in *C*. *sinensis*-infected WT and *TLR3*^*-/-*^ mice. The data were obtained from 30 mice in each group, and three independent repeated trials were conducted. ***p<0.001, T-tests and two-way ANOVA were performed to compare the samples.

RT-qPCR revealed a significant elevation of TLR3 mRNA in the liver of *C*. *sinensis-*infected WT mice at 7 dpi, 15dpi, and 35 dpi (Fig 1A). *C*. *sinensis*-infected *TLR3*^*-/-*^ mice showed lower weight and a reduced survival rate compared to *C*. *sinensis*-infected WT mice (Fig 1B and 1C). The survival rate of *C*. *sinensis*-infected *TLR3*^*-/-*^ mice was reduced by approximately 10% compared with that infected WT mice (Fig 1C). There was a significant difference in the number of intrahepatic parasites between *C*. *sinensis*-infected WT and *TLR3*^*-/-*^ mice, and the number of *C*. *sinensis* adults in *TLR3*^*-/-*^ mice was significantly higher than that in WT mice (Fig 1D).

### 2. TLR3 deficiency aggravated *C*. *sinensis*-induced bile duct lesions and liver inflammation

**Fig 2:**
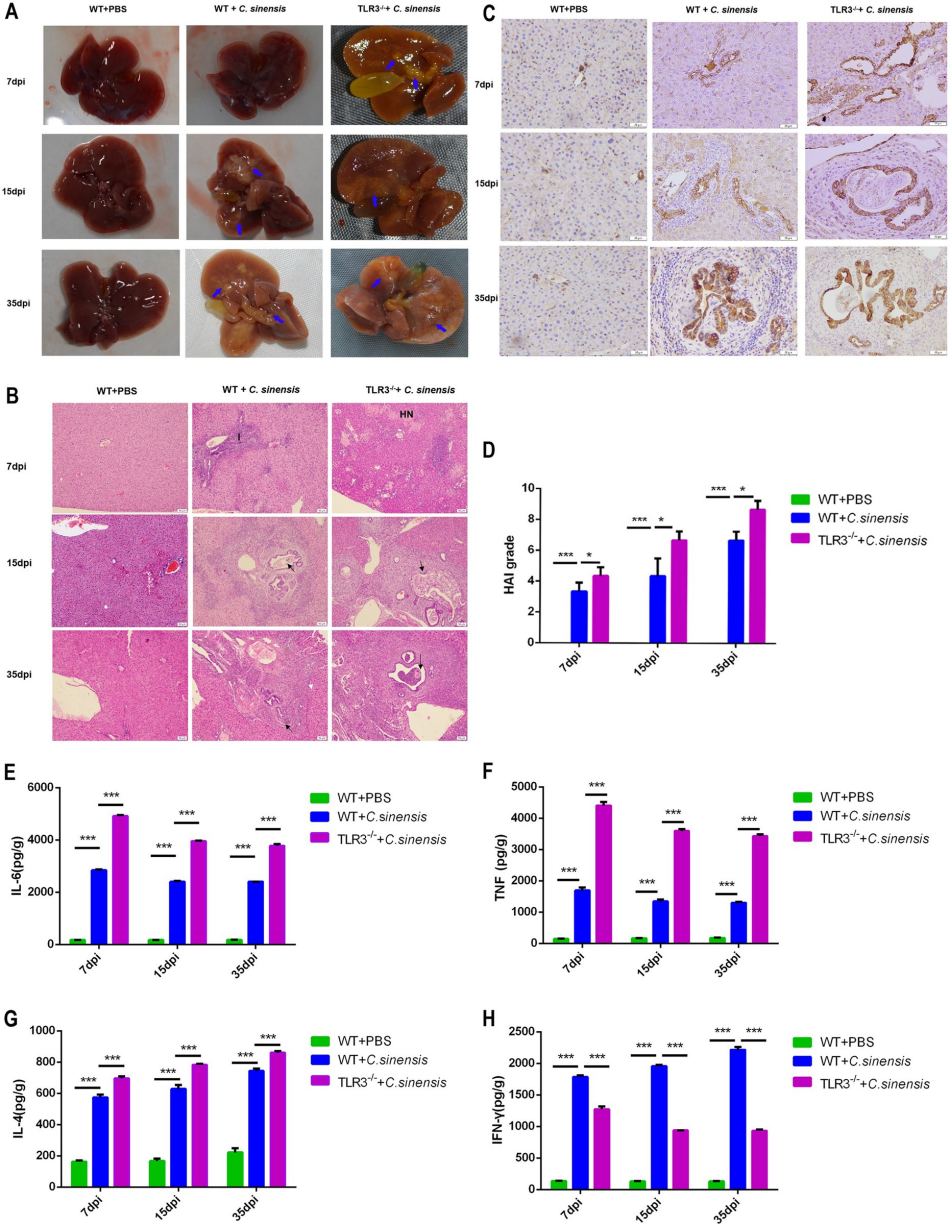
TLR3 deficiency exacerbated liver inflammation and biliary damage caused by *C*. ***sinensis* infection.** (A)Liver lesions of WT and *TLR3*^*-/-*^ mice after *C*. *sinensis*-infection were observed. *C*. *sinensis* infection caused hepatomegaly, sclerosis, jaundice and fibrous connective tissue hyperplasia. Blue arrow pointed to fibrous connective tissue hyperplasia. (B) Pathological changes of liver in *C*. *sinensis*-infected WT and *TLR3*^*-/-*^ mice were observed. Liver tissue sections were prepared and stained with H&E. The parasites in bile duct were indicated by black arrows. Hepatic cells necrotic was indicated by ‘HN’. Inflammatory cells were indicated by the letter ‘I’. Scale bars = 50 μm. (C) CK-19 in the liver of *C*. *sinensis*-infected WT and *TLR3*^*-/-*^ mice were tested by immunohistochemistry. The brown staining in the figure represented CK-19 positive. Scale bars = 20 μm. (D) The degree of liver inflammation and biliary injury was calculated by hepatic HAI. (E-H) The cytokines productions in liver of *C*. *sinensis*-infected WT and *TLR3*^*-/-*^ mice were detected by ELISA separately. (E) IL-6; (F) TNF; (G) IL-4; (H) INF-γ. The data were obtained from 30 mice in each group, and three independent repeated trials were conducted. *p<0.05, ***p<0.001, two-way ANOVA were performed to compare the samples.

Significant pathological changes, including hepatomegaly, cholestasis, jaundice, dilated protrusions of bile ducts, and connective tissue hyperplasia, were observed in the livers of *C*. *sinensis*-infected WT mice. TLR3 deficiency deteriorated the liver lesions induced by *C*. *sinensis*, with more pronounced bile duct degeneration, connective hyperplasia, and cholestasis than in WT mice (Fig 2A).

We observed pathological injury in the livers and bile ducts of *C*. *sinensis*-infected *TLR3*^*-/-*^ and WT mice at 7,15, and 35 dpi. There were hepatocellular necrosis foci, large numbers of inflammatory cells gathered around the necrotic foci and portal veins, cholangiectasis, and BECs proliferating and disorganized in the livers of *C*. *sinensis*-infected WT mice. Liver inflammation and biliary injury in *C*. *sinensis*-infected mice increased with infection duration (Fig 2B–2D). The livers of *C*. *sinensis*-infected *TLR3*^*-/-*^ mice showed more severe hepatic necrosis foci and bile duct degeneration, as well as more inflammatory cells compared to WT mice (Fig 2B–2D).

The expression levels of IL-6, IL-4, TNF and IFN-γ in livers of *C*. *sinensis-*infected WT mice were significantly increased compared to PBS treatment mice at 7,15, and 35 dpi (Fig 2E–2H). IL-6, TNF, and IL-4 were significantly increased in the livers of *TLR3*^*-/-*^ mice compared to WT mice (Fig 2E–2G). In contrast, IFN-γ expression was remarkably reduced in the livers of *TLR3*^*-/-*^ mice compared to that in *C*. *sinensis-*infected WT mice (Fig 2H).

### 3. TLR3 deficiency aggravated liver fibrosis caused by *C*. *sinensis*

**Fig 3:**
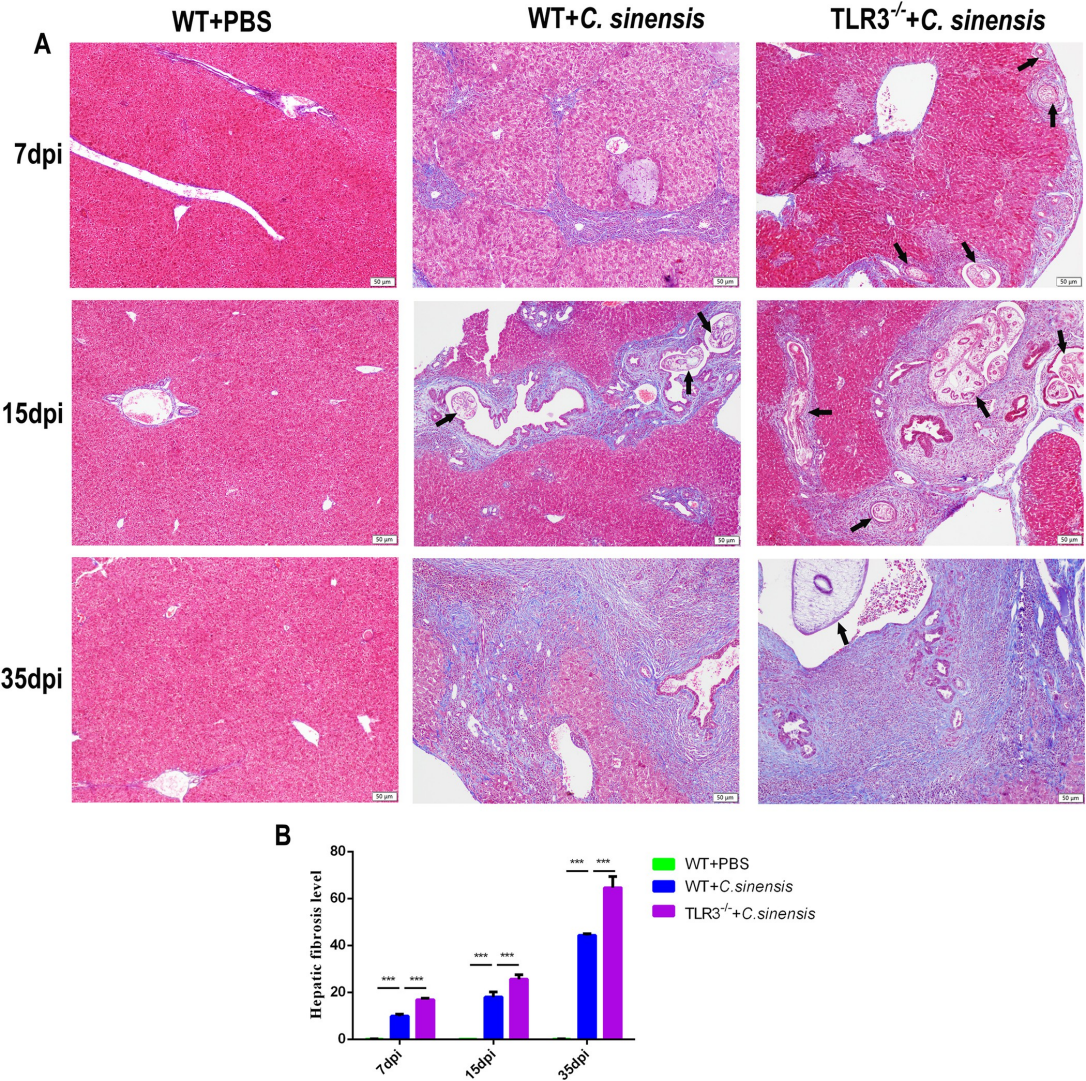
TLR3 deficiency exacerbated liver fibrosis caused by *C*. ***sinensis*.** (A)The collagen deposition in the liver of *C*. *sinensis*-infected mice were observed by Masson staining. Collagen deposition was visualized by blue stripes. The parasites were indicated by the black arrows. Scale bars = 50 μm. (B) Statistical analysis of the collagen positive distribution proportion was semi-quantified in liver of mice. The data were obtained from 30 mice in each group, and three independent repeated trials were conducted. ***p<0.001, two-way ANOVA were performed to compare the samples.

Liver fibrosis in *C*. *sinensis*-infected WT and *TLR3*^*-/-*^ mice was observed using Masson staining. The results showed that *C*. *sinensis* caused severe liver fibrosis, with persistent accumulation of collagen fibers around bile ducts at 7 dpi and 15 dpi, and even developed liver fibrosis at 35 dpi in WT mice (Fig 3A and 3B). The deposition of collagen fibrils in the liver of *TLR3*^*-/-*^ mice was more severe and more rapid at 7 dpi, 15 dpi, and 35 dpi than in WT mice (Fig 3A and 3B). These results suggest that TLR3 attenuates the liver fibrosis caused by *C*. *sinensis*.

### 4. TLR3 deficiency promoted the activation of TGF-β/Smad pathway and myofibroblasts induced by *C*. *sinensis*

**Fig 4:**
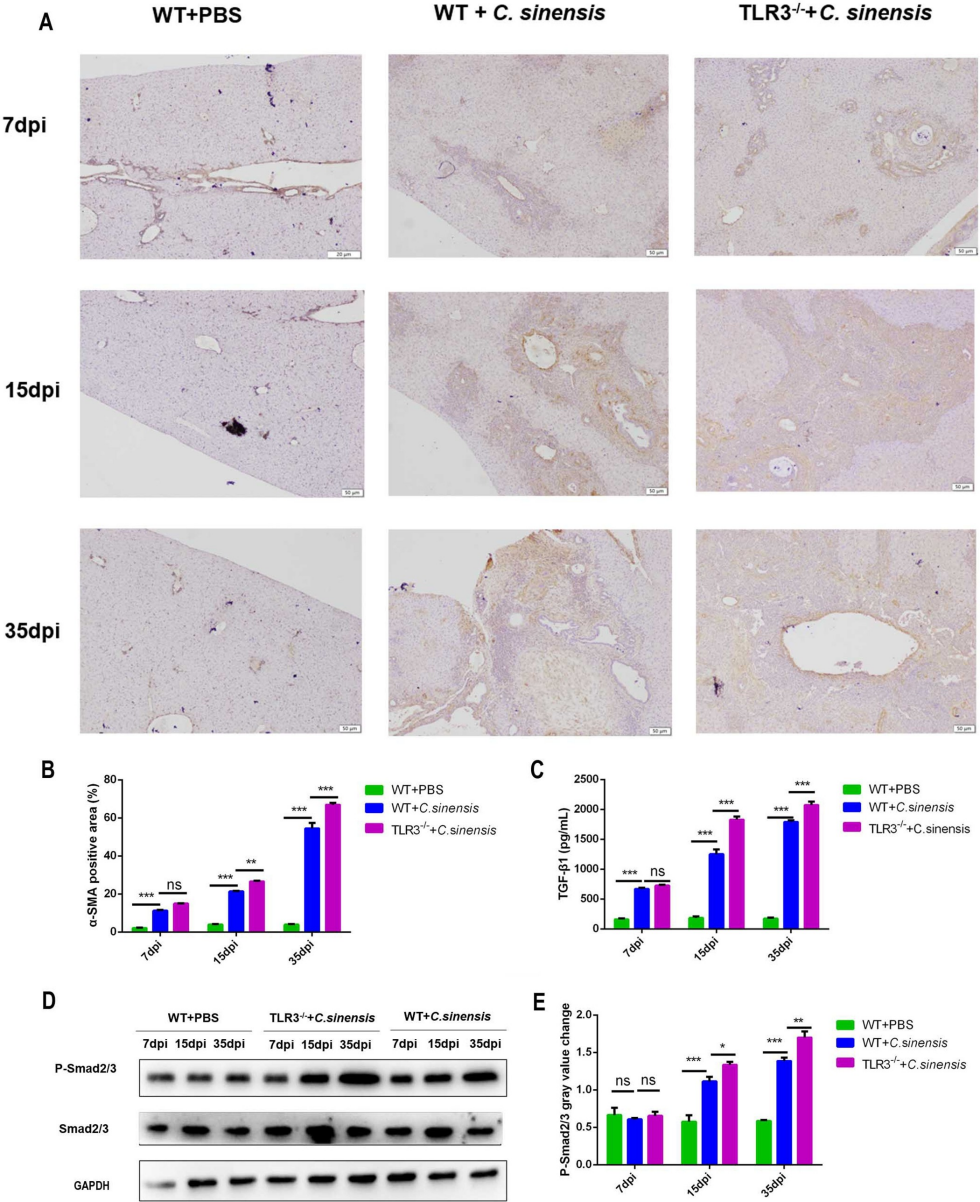
TLR3 deficiency promoted the activation of TGF-β1/Smad2/3 pathway and myofibroblast caused by *C*. ***sinensis*.** (A)The location and amount of myofibroblast distribution in the liver after *C*. *sinensis* infection were observed by α-SMA immunohistochemical staining. The yellow staining represented α-SMA positive. Scale bars = 50 μm. (B) Statistical analysis the α-SMA positive myofibroblasts proportion were semi-quantified in liver of mice. (C) TGF-β1 expression in the liver of *C*. *sinensis*-infected WT and *TLR3*^*-/-*^ mice was tested by ELISA. (D) The levels of samd2/3 phosphorylation in the liver of *C*. *sinensis*-infected WT and *TLR3*^*-/-*^ mice were detected by Western blot. (E) Gray analysis in Fig D. The data were obtained from 30 mice in each group, and three independent repeated trials were conducted. Ns *p*>0.05, *p<0.05, **p<0.01, ***p<0.001, two-way ANOVA were performed to compare the samples.

We examined myofibroblast activation in the livers of *C*. *sinensis*-infected mice via α-SMA immunohistochemistry. The results showed that α-SMA-positive myofibroblasts appeared around the bile duct at 7 dpi and progressively increased at 15 dpi and 35 dpi in the livers of *C*. *sinensis*-infected WT mice (Fig 4A and 4B). The number of positive myofibroblasts was significantly higher in the livers of *C*. *sinensis-*infected *TLR3*^*-/-*^ mice than that in *C*. *sinensis*-infected WT mice at 15 dpi and 35 dpi (Fig 4A and 4B).

The TGF-β/Smad pathway was detected in the livers of *C*. *sinensis-*infected mice at 7, 15, and 35 dpi. The results showed that *C*. *sinensis* infection significantly promoted TGF-β1 expression at 7, 15, and 35 dpi (Fig 4C) and increased the Smad2/3 phosphorylation at 15 and 35 dpi in the livers (Fig 4D and 4E). TLR3 deficiency further increased the expression of TGF-β1(Fig 4C) and phosphorylation level of Smad2/3 at 15 and 35 dpi (Fig 4D and 4E). These results suggest that host TLR3 contributes to reducing *C*. *sinensis-*induced the activation of TGF-β/Smad pathway and myofibroblast, thereby alleviating liver fibrosis.

### 5. CsEVs regulated the production of proinflammatory cytokines via TLR3-mediated p38 and ERK pathways

**Fig 5:**
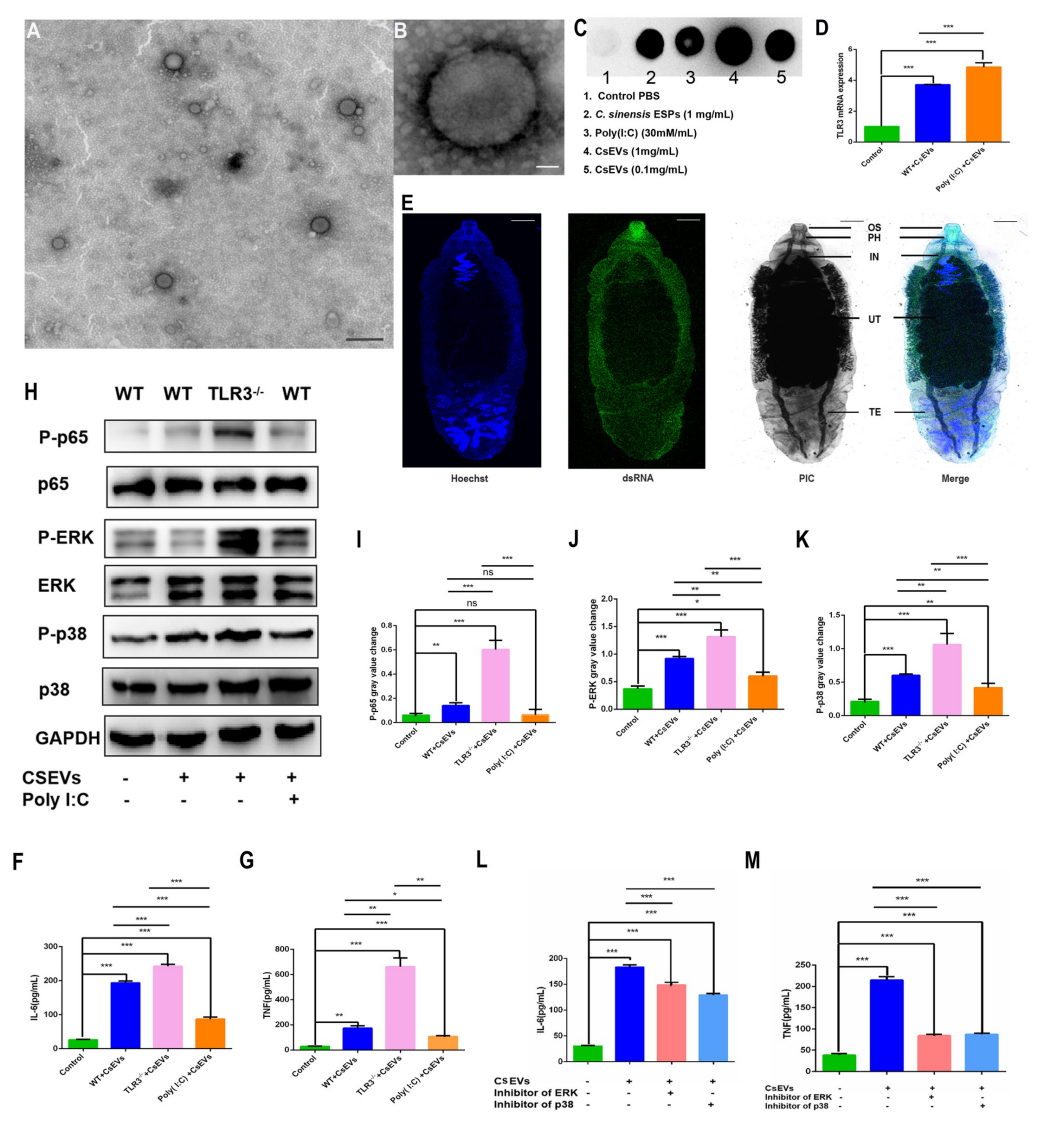
CsEVs activated BECs TLR3 and regulated the hyperphosphorylation of p38 and ERK to reduce IL-6 and TNF expression. (A,B) Transmission electron microscopic observed the CsEVs. (A) The size of the CsEVs was 80–120 μm in homogeneous particles. (B) Magnified CsEVs are dimensional and spherical, with a slight depression. (C) dsRNA detected in CsEVs using DIBA, CsESPs, and poly (I:C) was used as a positive control. (D) TLR3 expression in BECs challenged with CsEVs (50 μg/mL) and pretreated with poly (I:C) (30 μg /mL) were detected by RT-qPCR, the mRNA level was normalized to GAPDH. (E) dsRNA expression in *C*. *sinensis* was detected using immunofluorescence. The J2 antibody was used to perform dsRNA. Scale bars = 1mm. (F, G) Secretion levels of IL-6 and TNF in the supernatants of BECs. (H) The phosphorylation of p65, ERK, and p38 in BECs challenged with CsEVs (50 μg/mL) and pretreated with poly (I:C) (30 μg/mL) was analyzed by western blotting. (I-K) Gray analysis in Fig C. (L, M) IL-6 and TNF production in the supernatant of BECs, which were pretreated with or without p38 and ERK inhibitors for 1 h, and then co-incubated with CsEVs for 18 h, were measured by ELISA. Ns *p*>0.05, *p<0.05, **p<0.01, ***p<0.001, multiple T-tests were performed to compare the samples.

To explore the TLR3-mediated mechanism of *C*. *sinensis*-induced liver fibrosis, we isolated CsEVs and examined the phosphorylation of inflammatory pathways and cytokine expression in mouse BECs stimulated with CsEVs. The results showed that CsEVs were slightly concave and spherical in shape, with a size of approximately 80–120 nm (Fig 5A and 5B). CsEVs were rich in dsRNA and significantly activated the transcriptional level of TLR3 in BECs (Fig 5C and 5D). Further, rCsEVs co-incubated with BECs, RT-PCR detected the expression of TLR3 mRNA. The results showed that rCsEVs could significantly activate the expression of TLR3 in BECs, and there was no significant difference from untreated CsEVs co-incubation BECs (S1 Fig). We performed immunofluorescence staining of adults using the J2 antibody to determine the dsRNA distribution in *C*. *sinensis*. The results showed that high expression of dsRNA was distributed in the oral sucker, pharynx, gut, and body surface of *C*. *sinensis* (Fig 5E), confirming that *C*. *sinensis* could activate TLR3 via excreting dsRNA.

In addition, IL-6 (Fig 5F) and TNF (Fig 5G) expression increased significantly in CsEV-stimulated WT BECs. IL-6 (Fig 5F) and TNF (Fig 5G) expression was significantly increased in TLR3 deficient BECs co-stimulated with CsEVs compared to WT BECs. Moreover, IL-4 and IFN-γ were not detected in the incubated supernatants. Next, we explored the mechanism through which TLR3 regulates the release of proinflammatory factors induced by CsEVs. We found that the phosphorylation of p65 (Fig 5H and 5I), ERK (Fig 5H and 5J), and p38 (Fig 5H and 5K) was significantly higher in WT BECs than in cells without irritants, and further increased in TLR3^-/-^ BECs induced by CsEVs (Fig 5H–5K). Poly (I:C) pretreatment partially inhibited the phosphorylation of ERK (Fig 5H and 5J) and p38 (Fig 5H and 5K). P38 and ERK inhibitors were used to explore the regulatory mechanism of IL-6 and TNF expression, and the results showed that the inhibitors could significantly inhibit IL-6 (Fig 5L) and TNF (Fig 5M) expression induced by CsEVs, respectively.

### 6. TLR3 deficiency promoted the liver lesions and myofibroblasts activation induced by CsEVs

**Fig 6:**
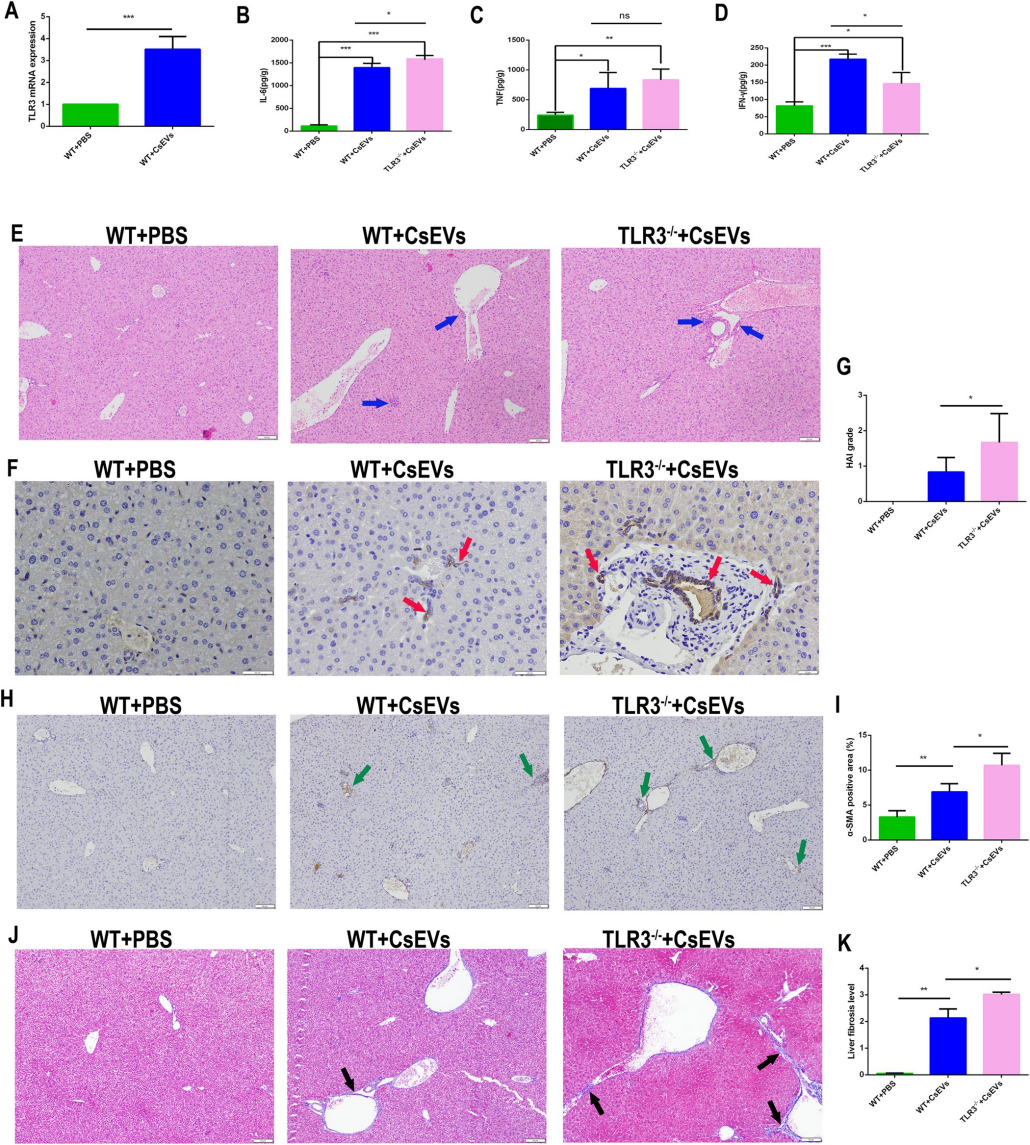
TLR3 deficiency promoted the liver injuries and myofibroblasts activation induced by CsEVs. (A) TLR3 expression in CsEVs injection mouse liver, the mRNA level was normalized to GAPDH. (B-D) Secretion levels of IL-6 and TNF in liver of CsEVs injection WT and *TLR3*^-/-^ mice. (E) Pathological changes of liver in CsEVs injected WT and *TLR3*^*-/-*^ mice were observed. Liver tissue sections were prepared and stained with H&E. Inflammatory cells were indicated by the blue arrow. Scale bars = 50 μm. (F) CK-19 in the liver of CsEVs injected WT and *TLR3*^*-/-*^ mice were tested by immunohistochemistry. The brown staining in the figure represented CK-19 positive and indicated by the red arrow. Scale bars = 20 μm. (G) The degree of liver inflammation and biliary injury was calculated by hepatic HAI. (H) The location and amount of myofibroblast distribution in the liver after CsEVs injection were observed by α-SMA immunohistochemical staining. The yellow staining represented α-SMA positive and indicated by the green arrow. Scale bars = 50 μm. (I) Statistical analysis the α-SMA positive myofibroblasts proportion were semi-quantified in liver of mice. (J) The collagen deposition in the liver of CsEVs injected mice were observed by Masson staining. Collagen deposition was visualized by blue stripes and indicated by the black arrow. Scale bars = 50 μm. (K) Statistical analysis of the collagen positive distribution proportion was semi-quantified in liver of mice. The data were obtained from 30 mice in each group, and three independent repeated trials were conducted. Ns *p*>0.05, *p<0.05, **p<0.01, ***p<0.001, multiple T-tests were performed to compare the samples.

To investigate whether TLR3 can be activated by CSEVs and participate in the pathogenesis of *C*. *sinensis*, we injected WT and *TLR3*^-/-^ mice with CsEVs. The CsEVs injection significantly increased the expression of TLR3 in the liver (Fig 6A) and promoted IL-6 (Fig 6B), TNF (Fig 6C) and IFN-γ expression (Fig 6D). In addition, CsEVs altered the bile duct morphology and induced inflammatory cells to accumulate, myofibroblasts activation and collagen expression in liver of WT mice (Fig 6E–6K). CsEVs result in higher IL-6 and TNF expression and lower IFN-γ expression in *TLR3*^-/-^ mice (Fig 6B–6D), accompanied by more serious liver injures (Fig 6E–6G), myofibroblast activation and collagen deposition (Fig 6H–6K).

### 7. Poly (I:C) intervention increased body weight and decreased the mortality and parasite burden due to *C*. *sinensis* infection in mice

**Fig 7:**
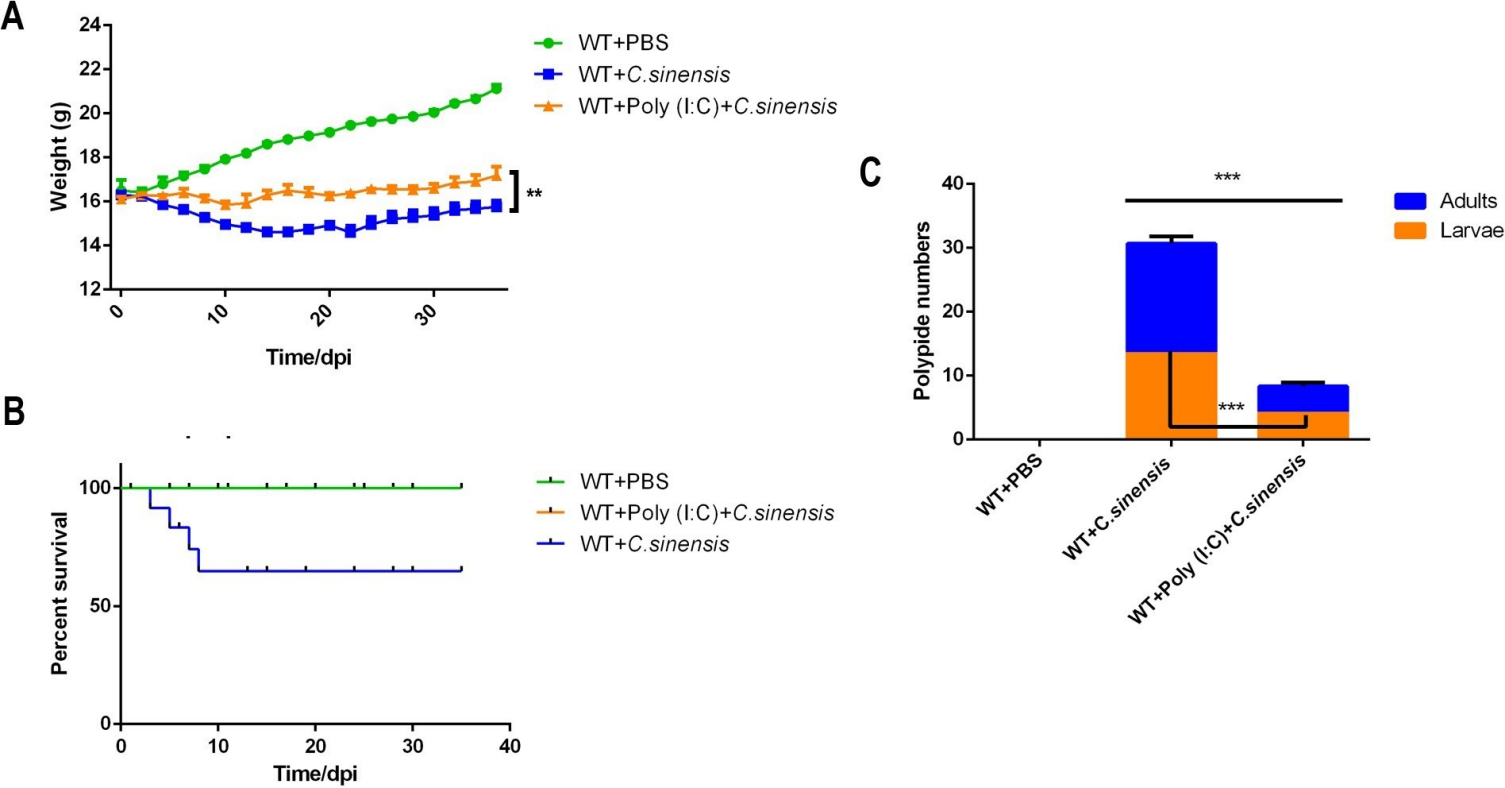
Therapeutic effect of poly (I:C) on *C*. ***sinensis*-infected mice.** WT mice with or without poly (I:C) intervention were infected with *C*. *sinensis*. (A) The weights of mice were recorded daily and plotted statistically. (B) Mortality of the mice was recorded and plotted daily after *C*. *sinensis* infection. (C) Number of intrahepatic parasites was counted. The data were obtained from 30 mice in each group, and three independent repeated trials were conducted. ***p<0.001, T-tests were performed to compare the samples.

Compared with *C*. *sinensis*-infected WT mice, poly (I:C) intervention significantly alleviated the weight loss in mice caused by *C*. *sinensis*-infection (Fig 7A). The mortality of poly (I:C) intervened *C*. *sinensis*-infection WT mice dropped by 0 from 40% (12/30) in the absence of poly (I:C) intervention in WT mice (Fig 7B). In addition, poly (I:C) intervention significantly reduced the number of intrahepatic parasites from an average of 29 parasites/mouse in WT-infected mice to an average of 8 parasites/mouse (Fig 7C), and the number of *C*. *sinensis* adults was significantly reduced (Fig 7C).

### 8. Poly (I:C) intervention reduced liver inflammation and proinflammatory cytokine release induced by *C*. *sinensis*

**Fig 8:**
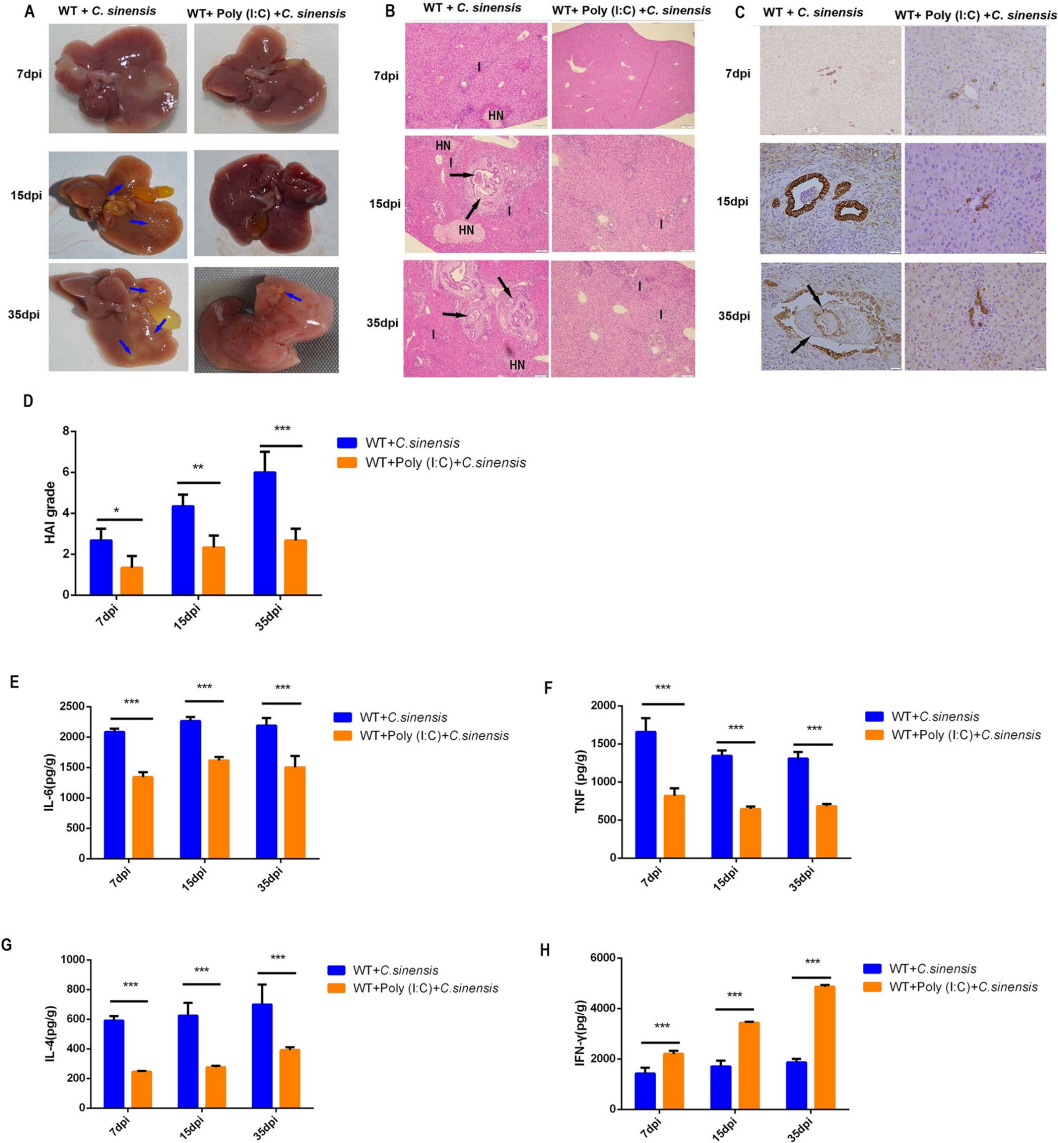
Poly (I:C) intervention presented significantly beneficial on the liver lesion, liver inflammation and biliary injury caused by *C*. ***sinensis*.** WT mice with or without poly (I:C) intervention were infected with *C*. *sinensis*. (A) Liver lesions were observed in the mice. Blue arrow indicates fibrous connective tissue hyperplasia. (B) Pathological changes were observed in the liver of mice. Parasites in the bile duct are indicated by black arrows. Hepatic cell necrosis is indicated by ‘HN’. Inflammatory cells were indicated by the letter ‘I’. Scale bars = 50 μm. (C) CK-19 expression in the liver of mice was tested by immunohistochemistry. Brown staining in the figure represents CK-19 positivity. Scale bars = 20 μm. (D) The degree of liver inflammation and biliary injury was calculated using the hepatic HAI. (E-H) Cytokine production in the mouse liver was detected separately by ELISA. (E) IL-6, (F) TNF, (G) IL-4, and (H) IFN-γ expression. The data were obtained from 30 mice in each group, and three independent repeated trials were conducted. *p<0.05, **p<0.01, ***p<0.001, two-way ANOVA were performed to compare the samples.

To investigate the effect of poly (I:C) on liver damage, pathological changes in the liver were observed, and proinflammatory cytokine production was measured. Compared with unintervened WT mice, poly (I:C)-intervened mice showed mini liver lesions (Fig 8A) with significantly reduced liver inflammation, BECs proliferation, and injury at 7, 15, and 35 dpi (Fig 8B–8D). The expression of pro-fibrotic cytokines, IL-6 (Fig 8E), TNF (Fig 8F) and IL-4 (Fig 8G) was significantly reduced, but IFN-γ (Fig 8H) was significantly increased in poly (I:C)-intervened mice compared to unintervened WT mice at 7, 15, and 35 dpi. These findings indicate that poly (I:C) intervention significantly alleviated liver inflammation and lesions caused by *C*. *sinensis*.

### 9. Poly (I:C) intervention alleviated liver fibrosis caused by *C*. *sinensis*

To investigate the role of poly (I:C) in liver fibrosis caused by *C*. *sinensis* in mice, we measured the activation of fibroblasts by α-SMA immunohistochemistry and collagen deposition with Masson staining at 7, 15, and 35 dpi.

**Fig 9:**
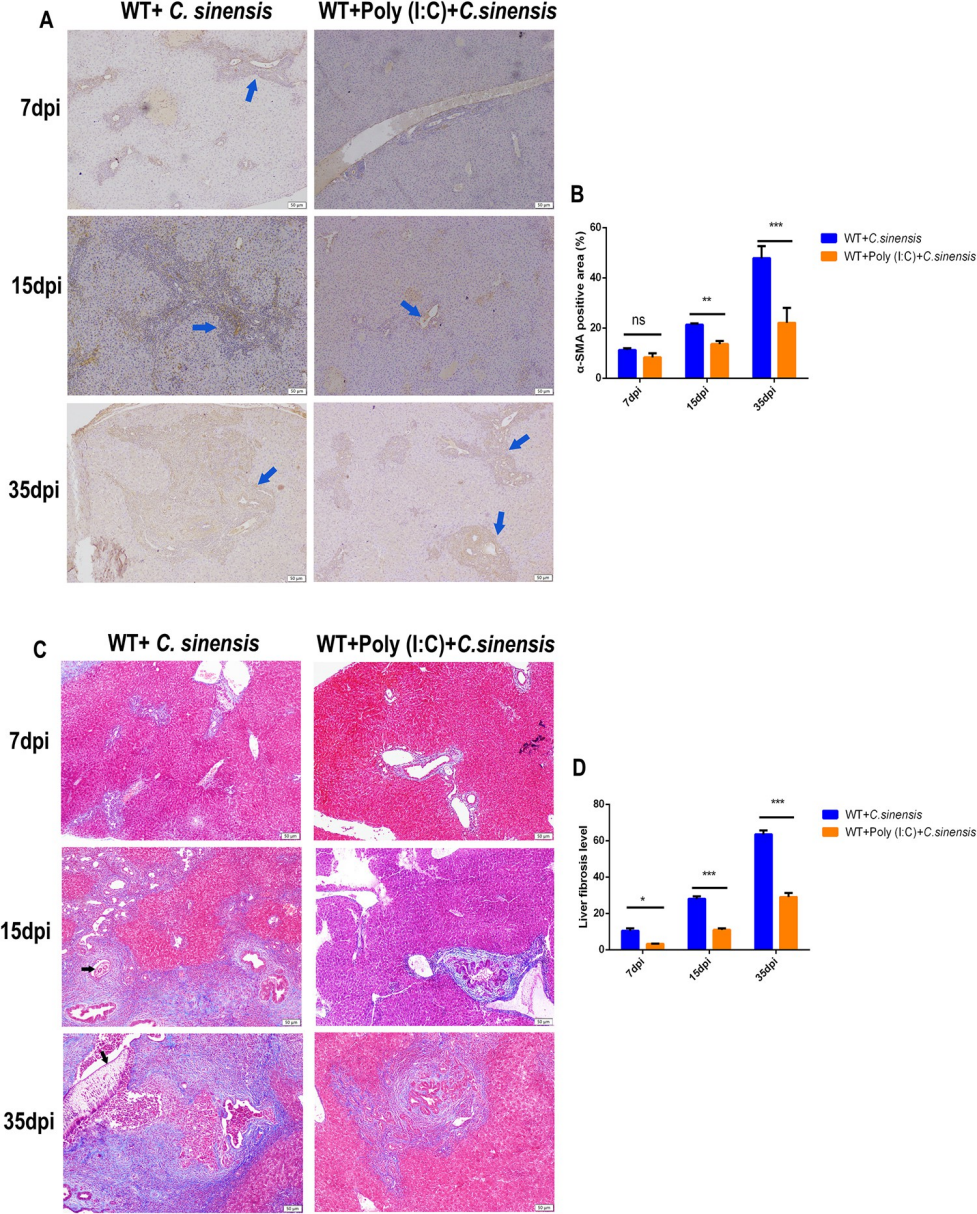
Poly (I:C) intervention reduced myofibroblast activation and collagen fibrosis deposition in *C*. ***sinensis-*infected mice.** WT mice with or without poly (I:C) intervention were infected with *C*. *sinensis*. Liver fibrosis of *C*. *sinensis*-infected mice at different time points was visualized using Masson staining and immunohistochemistry. (A) The location and distribution of myofibroblasts in the liver were observed via α-SMA immunohistochemical staining. Yellow staining in the figure represents α-SMA positivity. The parasites are indicated by black arrows. Scale bars = 50 μm. (B) Statistical analysis of the proportion of α-SMA-positive myofibroblasts was semi-quantified in the livers of mice. (C) The location of collagen fibers in the liver was determined by Masson staining. Collagen deposition was visualized using blue stripes. The parasites are indicated by black arrows. Scale bars = 50 μm. (D) Statistical analysis of the collagen-positive distribution proportion was semi-quantified in the livers of mice. The data were obtained from 30 mice in each group, and three independent repeated trials were conducted. Ns *p*>0.05, *p<0.05, **p<0.01, ***p<0.001, two-way ANOVA were performed to compare the samples.

The results showed that poly (I:C) intervention significantly reduced the number of myofibroblasts in *C*. *sinensis*-infected WT mice compared to poly (I:C)-unintervened mice (Fig 9A). Statistical analysis of the positive area of myofibroblasts showed that the number of myofibroblasts in the liver of poly (I:C)-intervened mice decreased by 7.76% and 25.73% at 15 and 35 dpi, respectively (Fig 9B).

Masson staining results showed that *C*. *sinensis*-induced collagen deposition in poly (I:C)-intervened WT mice was significantly reduced compared to that in poly (I:C)-unintervened mice (Fig 9C). Poly (I:C)-intervened mice showed almost no collagen deposition around the bile duct at 7 and 15 dpi (Fig 9C and 9D). Collagen fibrotic deposition in poly (I:C)-intervened mice was significantly reduced compared with that in poly (I:C)-unintervened WT mice at 35 dpi (Fig 9C and 9D). These results suggest that poly (I:C) intervention reduced the number of positive myofibroblasts and collagen deposition caused by *C*. *sinensis* in mice.

## Discussion

TLR activation provides the first line of defense in the anti-pathogen immune response. The role of TLR3 in liver diseases promptes us to investigate whether it is involved in the liver fibrosis process caused by *C*. *sinensis* [21–23]. To elucidate the potential mechanism of TLR3 in *C*. *sinensis*-induced liver fibrosis, liver fibrosis modeling assays were performed using TLR3-deficient or normal C57/BL6 mice in our research. The experimental data from both types of mice helped us to explore the role of TLR3 in liver fibrosis caused *C*. *sinensis* and the mechanism of host-parasite interaction. It is noteworthy that TLR3 deficiency caused severe clonorchiasis with lower survival quality, higher liver damage, and more severe liver fibrosis. This study suggests that TLR3-based treatments have a great potential for applications in *C*. *sinensis* and other parasitic liver fibrosis.

EVs produced by *C*. *sinensis*, carrying parasite information, activating the innate immune response of mouse macrophages and BECs, produce proinflammatory cytokines [9,15]. The bile ducts are the normal parasitic site of *C*. *sinensis*, and the immune response of BECs TLRs play the important role during *C*. *sinensis* infection [9,24]. We used CsEVs and BECs to simulate the interaction process between *C*. *sinensis* and host *in vitro*, and found that *C*. *sinensis* releases large amounts of dsRNA via CsEVs, which were recognized by host TLR3 and activated the innate immune response.

The deregulation of response of TLRs to PAMPs in BECs can lead to various liver diseases [25]. The IL-6 and TNF secreted by BECs and macrophages are the vital cytokines in *C*. *sinensis* induced liver injure and fibrosis [15,24]. The present data indicated that BECs TLR3 deficiency resulted in significantly increased IL-6 and TNF secretion induced by CsEVs, which may be an important reason for more severe liver damage and liver fibrosis in *TLR3*^*-/-*^ mice infected with *C*. *sinensis*. And this inference was confirmed in the CsEVs injection mice. TLR3^-/-^ mice injection with CsEVs resulted in higher IL-6 and TNF expression, leading to more severe biliary injures.

On the other hand, the infection intensity of helminths is closely related to the severity of liver lesions caused by them [26]. We found that an important result of TLR3 deficiency was a significantly higher number of infected worms, which may be another important reason for more severe liver damage and liver fibrosis in *TLR3*^*-/-*^ mice infected with *C*. *sinensis*.

*C*. *sinensis* infection significantly activates the TGF-β/Smad pathway, leading to liver fibrosis [7,27]. The recombinant worm proteins rCsMF6p/HDM promote immune response and cell differentiation through the MAPK pathway [28]. However, the interaction between TGF-β/Smad and p38 in liver fibrosis induced by *C*. *sinensis* has not been clarified. Isorhamnetin protects against liver fibrosis via inhibition of TGF-β1-mediate Smad3 and p38 MAPK signaling pathways [29]. Drug-containing serum of rhubarb-astragalus reduce the protein expression of TGF-β1 and p38 MAPK and mRNA expression of SMA-α, Smad2 and Smad3 in HK-2 cells caused by the increase of TGF-β1, and the same results are found in the treatment of p38 inhibitors[30]. These studies indicate that TGF-β/Smad pathway and p38 pathway have the mutual regulatory role in the regulation of the epithelial-mesenchymal transformation and liver fibrosis. Our study clarified the mechanism by which TLR3-p38/ERK regulated cytokine expression promoted inflammation and damage, and also found that TLR3 deletion leaded to increased activation of TGF-β/Smad pathway in *C*. *sinensis*-induced liver fibrosis. We infer that TLR3-p38/ERK regulated cytokine expression may be one of the factors inducing TGF-β/Smad pathway activation which needs to be further explored in future research.

Based on this concept, we achieved promising results with TLR3 agonists for the treatment of *C*. *sinensis*. Poly (I:C), a viral dsRNA mimetic, is the most commonly used TLR3 [31]. In parasite control, poly (I:C) is used as a vaccine adjuvant to induce a multifunctional CD4^+^ T cell response and enhance antibody production against *Plasmodium falciparum* [32,33]. Total *Leishmania* antigens-poly (I:C) immunization resultes in good protection in mice, which is associated with decreased footpad swelling, histopathological alterations in the footpads, and parasite burdens [31]. There is no evidence that poly (I:C) can be used to control or treat liver flukes. In this study, we used poly (I:C) to treat liver fibrosis caused by *C*. *sinensis*. Poly (I:C) intervention significantly blocked the acute phase of death in mice, reduced the number of intrahepatic parasites, and alleviated *C*. *sinensis*-induced liver fibrosis, which demonstrated that poly (I:C) has great potential for application in the treatment of clonorchiasis caused by *C*. *sinensis*. *Opisthorchis viverrini* infection induces liver fibrosis and even cholangiocarcinoma, causing the serious disease burden in Southeast Asian countries [34]. Whether the positive contribution of poly (I:C) is also applicable to the pathogenic process of *O*. *viverrini* deserves further investigation in future studies.

**Fig 10:**
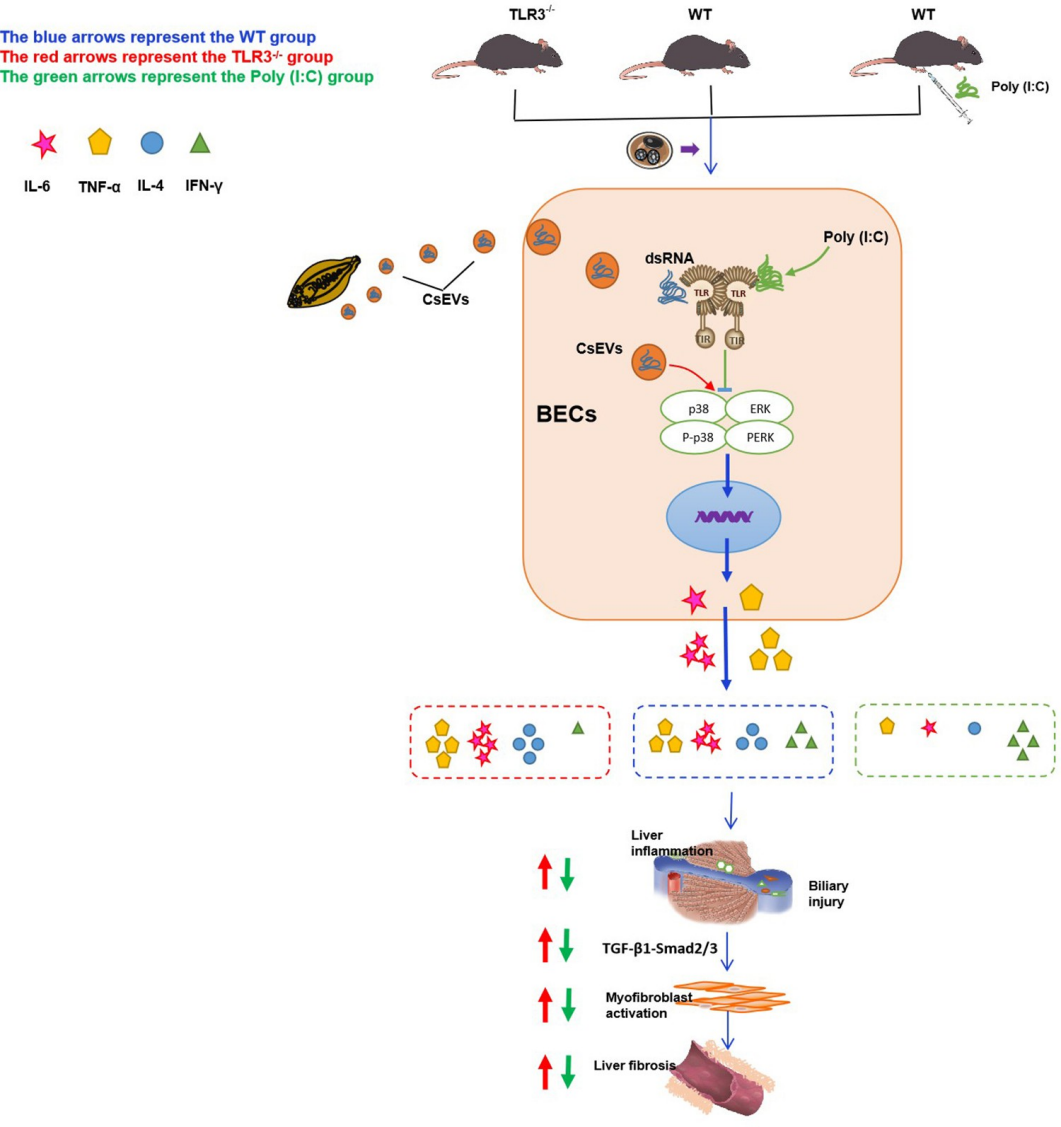
TLR3 inhibit IL-6 and TNF production via p38/ERK signaling pathways alleviate C. sinensis-induced liver fibrosis.

In summary, a new role for TLR3 in controlling *C*. *sinensis*-induced liver fibrosis was identified (Fig 10). TLR3 deficiency resulted in severe clonorchiasis in *C*. *sinensis*-infected mice compared with WT mice. Poly (I:C) is a promising drug for clonorchiasis treatment caused by *C*. *sinensis*. Our results will help in understanding the molecular mechanisms governing the host’s immune responses to *C*. *sinensis* infections and provide new information about clonorchiasis treatment.

## Supporting information

S1 Fig TLR3 expression in BECs challenged with CsEVs (50 μg/mL) and rCsEVs (50 μg/mL). TLR3 expression were detected by RT-qPCR, the mRNA level was normalized to GAPDH.
(TIF)

S1 Table Details of the antibodies used in this study. (DOCX)

S1 Data Excel spreadsheet containing, in separate sheets, the underlying numerical data and statistical analysis for Figure panels 1A-1D, 2D-2H, 3B, 4B, 4C, 4E, 5D, 5F, 5G, 5I-5M, 6A-6D, 6G, 6I, 6K, 7A-7C, 8D-8H, 9B, 9D and S1. (XLSX)
