# Supplementary material for: TLR3 activation by Clonorchis sinensis infection alleviates the fluke-induced liver fibrosis
Source: PLoS Negl Trop Dis. 2023 May 11;17(5):e0011325. doi: 10.1371/journal.pntd.0011325 (PMC10174496; doi:10.1371/journal.pntd.0011325)
Supplement: S1 Table — (DOCX) [file pntd.0011325.s002.docx]

**Table S1. Details of the antibodies used in this study.**

| **Antibodies** | **Description** | **Isotype** | **Dilution ratio** | **manufacturer** |
| --- | --- | --- | --- | --- |
| **p65** | monoclonal | Rabbit IgG | 1:1000 | Cell Signaling Technology  (Shanghai, China  ) |
| **Phospho-p65** | Rabbit monoclonal | Rabbit IgG | 1:1000 | Cell Signaling Technology |
| **Smad2/3** | Rabbit polyclonal | Rabbit IgG | 1:1000 | Abcam (Cambridge, USA  ) |
| **Phospho-Smad2/3** | Rabbit polyclonal | Rabbit IgG | 1:1000 | Abcam |
| **P38** | Rabbit monoclonal | Rabbit IgG | 1:1000 | Cell Signaling Technology |
| **Phospho-p38** | Rabbit monoclonal | Rabbit IgG | 1:1000 | Cell Signaling Technology |
| **ERK** | Rabbit monoclonal | Rabbit IgG | 1:1000 | Cell Signaling Technology |
| **Phospho-ERK** | Rabbit monoclonal | Rabbit IgG | 1:1000 | Cell Signaling Technology |
| **GAPDH** | Rabbit monoclonal | Rabbit IgG | 1:1000 | Cell Signaling Technology |
| **J2** | mouse monoclonal | Mouse IgG | 1:1000 | SCICONS (Susteren, Netherlands) |
| **α-SMA** | Rabbit monoclonal | Rabbit IgG | 1:400 | Abcam |
| **CK-19** | Rabbit monoclonal | Rabbit IgG | 1:400 | Abcam |
| **HRP-link-**  **antibodies** |  | Goat-anti-  rabbit IgG | 1:5000 | Proteintech (Wuhan, China) |
|  |  | Rabbit-anti-mouse  IgG | 1:5000 | Proteintech |
| **FITC-link-**  **antibodies** |  | Goat Anti-Mouse | 1:400 | Proteintech |
